# Polycarbonate–Acrylonitrile Butadiene Styrene Three Dimensional Printing Material Exhibits Biocompatibility and Enhances Osteogenesis and Gingival Tissue Formation with Human Cells

**DOI:** 10.3390/cells14030167

**Published:** 2025-01-22

**Authors:** Li Xiao, Naohiro Shimamura, Takashi Kamio, Ryoji Ide, Mai Mochizuki, Taka Nakahara

**Affiliations:** 1 Department of Physiology, School of Life Dentistry at Tokyo, The Nippon Dental University, 1-9-20 Fujimi, Chiyoda-ku, Tokyo 102-8159, Japan; ryo-ide@tky.ndu.ac.jp; 2 Department of Dental Anesthesiology, School of Life Dentistry at Tokyo, The Nippon Dental University, 1-9-20 Fujimi, Chiyoda-ku, Tokyo 102-8159, Japan; shimamura@tky.ndu.ac.jp; 3 Department of Oral and Maxillofacial Radiology, School of Life Dentistry at Tokyo, The Nippon Dental University, 1-9-20 Fujimi, Chiyoda-ku, Tokyo 102-8159, Japan; kamio@tky.ndu.ac.jp; 4 Department of Developmental and Regenerative Dentistry, School of Life Dentistry at Tokyo, The Nippon Dental University, 1-9-20 Fujimi, Chiyoda-ku, Tokyo 102-8159, Japan; mai-m@tky.ndu.ac.jp (M.M.); t.nakahara@tky.ndu.ac.jp (T.N.); 5 Department of Life Science Dentistry, The Nippon Dental University, 1-9-20 Fujimi, Chiyoda-ku, Tokyo 102-8159, Japan

**Keywords:** 3D-printing technology, biocompatibility, surgical guide resin, PEEK, PC-ABS, osteogenesis, gingival tissue equivalents, culture insert, SHED

## Abstract

Three dimensional (3D) printing materials are widely used in dental applications, but their biocompatibility and interactions with human cells require evaluation. This study aimed to identify materials meeting biocompatibility, mechanical strength, and tissue-forming requirements for safe dental applications. We assessed the cytotoxicity of resins and thermoplastic filaments in human HaCaT keratinocytes, gingival fibroblasts (hGFs), and stem cells from human exfoliated deciduous teeth (SHED) using PrestoBlue assays. Three resins, including two types of surgical guide resins, exhibited strong cytotoxicity after 4–72 h, while 2 h exposure to an FDA-approved surgical guide resin did not affect SHED cell viability. In contrast, six thermoplastic filaments showed no significant cytotoxicity even after 72 h. Among these, polycarbonate–acrylonitrile butadiene styrene (PC-ABS) demonstrated excellent toughness, heat resistance, and surface quality at a low cost. SHED cells cultured on PC-ABS dishes and micro bone structures showed strong proliferation and osteogenic potential. Culture inserts made of PC-ABS also supported the growth of HaCaT keratinocytes and the hGFs formed gingival tissue, which was superior to that formed on commercially available PET inserts. In conclusion, PC-ABS is a promising 3D printing material for dental applications due to its biocompatibility, ability to promote osteogenesis, and support for gingival tissue formation, with no observed cytotoxicity.

## 1. Introduction

Three-dimensional (3D) printing technology has revolutionized the field of dentistry by enabling the precise fabrication of dental prostheses, implants, surgical guides, and tissue engineering scaffolds [1,2]. The flexibility to design complex geometries and tailor materials to clinical needs makes 3D printing a transformative tool in personalized dental care. However, the safety and biocompatibility of 3D-printed materials remain critical concerns that must be addressed before widespread clinical implementation [3,4].

Resins and thermoplastic filaments are the two primary types of 3D printing materials currently used in dental applications. Resins, especially those approved by regulatory organizations such as the FDA, are often employed in fabricating surgical guides and dental models [5,6]. Despite their widespread use, resin-based materials can release cytotoxic components during polymerization, posing potential risks to the surrounding tissues and limiting their application for long-term biological use [7]. In contrast, thermoplastic filaments, such as polyetheretherketone (PEEK), offer advantages such as lower toxicity, higher mechanical strength, and improved biocompatibility [8,9].

Recent studies have highlighted the need for 3D printing materials that not only meet mechanical and manufacturing requirements but also support cellular viability, tissue regeneration, and osteogenesis [10,11]. Polycarbonate–acrylonitrile butadiene styrene (PC-ABS), a thermoplastic filament, is gaining attention due to its superior mechanical properties, thermal resistance, and cost-effectiveness [12]. However, its biological compatibility and capacity to promote tissue formation in dental applications remain largely unexplored.

This study aimed to evaluate the biocompatibility of various 3D printing materials, including resins and thermoplastic filaments, by assessing their cytotoxicity in three types of human cells. Additionally, we examined the potential of PC-ABS to support cell proliferation, osteogenesis, and tissue formation, which are essential properties for its application in regenerative dentistry.

## 2. Materials and Methods

### 2.1. 3D Printers

The 3D printers used for this study are: 1. LCD (liquid crystal display) 3D printer for resin sculptures: ELEGOO Mars 3 Pro (ELEGOO, Shenzhen, China). 2. Four types of FDM (fused deposition modeling) 3D printers for filament sculptures: FUNMAT HT Enhanced DESIGNED FOR PEEK 3D PRINTING (INTAMSYS Technology Co., Ltd., Shanghai, China), Qidi Tech X-Plus 3 (Qidi Tech. Shenzhen, China), Value3D MagiX MF-2500EPII and Value3D MagiX MF-800 (MUTOH INDUSTRIES, Ltd., Tokyo, Japan).

### 2.2. 3D Printing Materials

The 3D printing materials used in this study, along with their properties, are detailed in Table 1 according to the manufacturer’s specifications. All sculptures, including those fabricated from PLA (polylactide) and PETG (polyethylene terephthalate glycol), were thoroughly washed and sterilized by autoclaving at 121 °C for 20 min before use in cell culture. While all sculptures maintained their shapes after a single autoclaving cycle, repeated autoclaving (2–3 cycles) revealed differences in material durability. Products made from heat-resistant materials retained their shapes and properties, whereas PLA and PETG products became deformed and tattered.

### 2.3. Reagents

Cell culture media (including MEM-α and DMEM media), Gibco^®^ GlutaMAX™ Supplement, and KnockOut™ serum replacement (KSR) were purchased from Thermo Fisher Scientific, Tokyo, Japan. Dexamethasone (194561), disodium β-glycerophosphate pentahydrate (048-34332), L-ascorbic acid 2-phosphate trisodium salt (019-28472), antibiotic–antimycotic solution (161-23181) and other reagents were purchased from FUJIFILM Wako Chemical Co., Osaka, Japan.

### 2.4. Cell Culture

Human gingival fibroblasts (hGFs) were isolated from gingival tissues of healthy patients (aged 17–26) who underwent wisdom tooth extraction at Nippon Dental University Hospital, Tokyo. Stem cells from human exfoliated deciduous teeth (SHED) were isolated from deciduous incisors of a 7-year-old child at the same hospital. Both procedures were conducted under ethical guidelines approved by the Committee of Ethics, Nippon Dental University School of Life Dentistry, Tokyo (authorization number: NDU-T2019-01). Both hGFs and SHED were maintained in MEM-α supplemented with 10% fetal bovine serum (FBS), 1% antibiotic–antimycotic solution, and 1% Gibco^®^ GlutaMAX™ supplement [13,14].

Human immortalized skin epidermal keratinocytes (HaCaT) were generously provided by Professor Norbert E. Fusenig [15]. HaCaT cells were maintained in DMEM supplemented with 10% FBS, 1% antibiotic–antimycotic solution, and 1% Gibco^®^ GlutaMAX™ supplement.

All cells were incubated at 37 °C in a humidified environment containing 5% CO_2_ and 95% O_2_.

### 2.5. Cell Viability Assay

Cell viability was measured using a PrestoBlue^®^ solution (A13261, Thermo Fisher Scientific) following the manufacturer’s protocol. This assay relies on the reduction of the resazurin-based dye in PrestoBlue by metabolically active cells, producing a fluorescent resorufin signal proportional to cell viability. Fluorescence intensity was quantified using a microplate reader (SH-9000Lab, HITACHI) with excitation/emission wavelengths of 560 nm/590 nm, as described in our previous report [13].

### 2.6. Osteogenic Differentiation

SHED cells (3 × 10^4^ cells/well) were seeded on 3D-printed PC-ABS culture dishes (outer diameter = 1 cm) and micro bone structures (0.6 × 0.6 × 0.12 cm) placed in 24-well plates and cultured for 3–4 days. Once the cells reached 100% confluence, the medium was replaced with an osteogenic medium (MEM-α, 10% FBS, 1% GlutaMAX™ Supplement, 50 mM ascorbic acid 2-phosphate, 0.1 mM dexamethasone, and 10 mM β-glycerophosphate). The cultures were maintained in osteogenic conditions for an additional 2–3 weeks. Control cells were cultured in the maintenance medium under the same conditions. At the end of the cultivation period, the cells were fixed with 10% formalin neutral buffer solution and subsequently subjected to Giemsa, ALP and Alizarin red S staining.

### 2.7. Quantitation of Mineralization

We developed a novel method to quantify ALP staining. After staining the cells with an ALP staining kit (15682, Muto Pure Chemicals Co., Ltd., Osaka, Japan), 2N NaOH/DMSO (1:1) was added to each culture well and shaken for 10 min. The extract exhibited a purple color with an absorption peak at 530 nm. The extract was then transferred to a 96-well plate and its absorbance was measured with a microplate reader (HITACHI, SH-9000Lab) at 530 nm. Quantitation of Alizarin red staining followed a method similar to our previous report [16]. The absorbance of the extract from cells stained with Alizarin red was measured at 560 nm.

### 2.8. Preparation of 3D Gingival Tissue Equivalents (GTE)

The GTE were prepared using an improved method based on our previous research [13] and cultured in our customized XL-inserts [17] printed with PC-ABS filament. The negative control GTE was cultured in commercially available PET-inserts (353095, CORNING, Tokyo, Japan) under the same conditions. Briefly, hGFs were mixed with collagen type I-A gel (Nitta Gelatin, Osaka, Japan) and placed on an atelocollagen sheet (Integrin^®^ sheet, KOKEN Co., Ltd., Tokyo, Japan) in the XL-insert in maintenance medium. After 2–3 days, HaCaT keratinocytes were seeded on top of the hGFs–collagen gel mixture. One day later, the medium was replaced with KSR medium (MEM-α supplemented with 15% KSR, 1% antibiotic–antimycotic solution, and 1% Gibco^®^ GlutaMAX™ supplement) containing 5% FBS. After 1–3 days, the medium was replaced with KSR medium containing 1% FBS. Subsequently, after another 1–3 days, the medium was replaced with KSR medium without FBS, and the culture was maintained for 3–4 weeks.

At the end of the cultivation, the tissues were fixed in 10% formalin neutral buffer solution at 4 °C overnight. The GTE were then dehydrated, embedded in paraffin, sectioned, and stained with H&E for histological analysis.

### 2.9. Statistical Analysis

Statistical analyses were conducted following the methods outlined in our previous report [17]. Data were analyzed using GNU PSPP Statistical Analysis Software (version 0.8.2 gad9374) (https://www.gnu.org/software/pspp/, accessed on 1 November 2024) and EZAnalyze, an Excel-based tool (http://www.ezanalyze.com/, accessed on 1 November 2024). One-way analysis of variance (ANOVA) was performed, followed by Tukey’s test and Bonferroni correction as post hoc tests. Statistical significance was set at *p* < 0.05. Each experiment was independently repeated 3–5 times.

## 3. Results

### 3.1. Completion of 3D-Printed XL-Insert Part Using Different Materials

**Figure 1:**
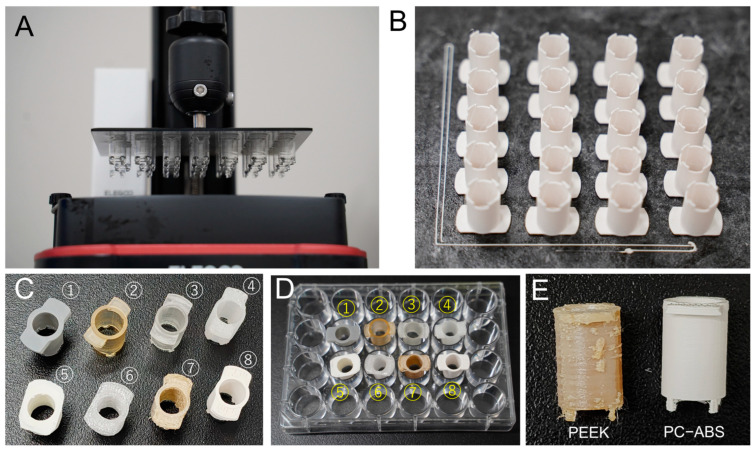
Photographs of the 3D-printed components of XL-inserts. The STL design file can be found in Supplemental File S1. (**A**) Components of XL-insert printed using an LCD 3D printer with zSG resin, shown still attached to the build plate. (**B**) XL-insert components printed using an FDM 3D printer with PC-ABS filament, with dimensions of 11 mm (*X*-axis), 18 mm (*Y*-axis), and 18 mm (*Z*-axis). (**C**) Components of XL-insert fabricated with various materials: ① ABS-like resin, ② SG resin, ③ zSG resin, ④ Nylon680 filament, ⑤ PLA filament, ⑥ PC filament, ⑦ PEEK filament, and ⑧ PC-ABS filament. (**D**) Components of XL-insert placed in a 24-well plate for scale and reference. (**E**) Comparison of XL-insert components made from PEEK and PC-ABS materials.

We previously developed an XL-insert for cell and tissue culture [17]. In this study, we refined the XL-insert using 3D printing technologies. One component of the XL-insert was designed using Blender 3.6 software (Supplementary File S1). Initially, an LCD 3D printer was employed to fabricate the component using three types of resins, including two surgical guide resins (Table 1). However, all resin-based constructs demonstrated cytotoxicity toward human cells, as evidenced by the results presented later. Consequently, we switched to thermoplastic filaments and utilized FDM printers for fabrication (Table 1). As shown in Figure 1, XL-insert components printed with eight different materials demonstrated varying levels of completion. Among these, sculptures made from PEEK (fabricated using the FUNMAT HT Enhanced printer, designed for PEEK 3D printing) and PC-ABS (fabricated using the Qidi Tech X-Plus 3 printer) closely matched the designed dimensions. Compared to PEEK, PC-ABS sculptures exhibited superior completion quality at a significantly lower cost.

### 3.2. Effects of 3D-Printed XL-Insert Components Fabricated from Different Materials on Cell Viability of HaCaT Keratinocytes and hGFs

**Figure 2:**
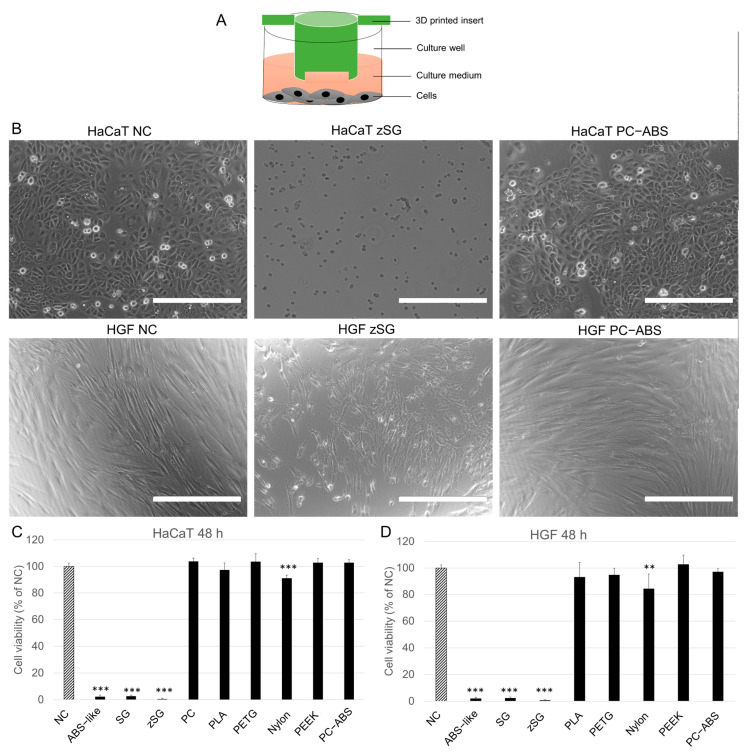
Cytotoxicity to HaCaT keratinocytes and hGFs of 3D-printed XL-insert components fabricated from different materials. HaCaT cells (3 × 10^4^ cells/well) or hGFs (1 × 10^4^ cells/well) were seeded in 24-well plates and cultivated for 24 h. The 3D-printed XL-insert components were then placed in the culture wells above the cells, and cultivation continued for an additional 48 h. At the end of the cultivation period, cell viability was measured using PrestoBlue assays. (**A**) Schematic illustration of the culture model. (**B**) Representative images of HaCaT cells and hGFs cultured with 3D-printed inserts fabricated from zSG resin and PC-ABS filament for 48 h. NC, negative control. Scale bars = 150 μm (**C**) Cell viability of HaCaT cells. (**D**) Cell viability of hGFs. ** *p* < 0.01, *** *p* < 0.001 vs. NC. Experiments were repeated five times independently.

We then evaluated the cytotoxicity of 3D-printed XL-insert components fabricated from nine different materials on HaCaT cells and hGFs using the PrestoBlue assay. After 48 h of exposure, all resin-based sculptures demonstrated strong cytotoxicity to both HaCaT cells and hGFs. As shown in Figure 2B, sculptures fabricated from FDA-approved zSG resin nearly eliminated all HaCaT cells and hGFs, while the negative control (NC) cells and PC-ABS-treated cells displayed healthy morphology. In contrast to resins, thermoplastic filaments generally showed no significant cytotoxicity toward HaCaT cells and hGFs, except for the FDA-approved Nylon 680 filament, which caused a mild reduction in cell viability in both HaCaT cells and hGFs (*p* < 0.001 and *p* < 0.01, respectively) (Figure 2C,D). 

### 3.3. Effects of 3D-Printed XL-Insert Components Fabricated from Different Materials on SHED Cell Viability

SHED cells, known for their multipotency, can differentiate into osteoblasts and contribute to bone regeneration and mineralization [18]. Studies suggest that SHED cells exhibit increased sensitivity to dental bioceramic materials, showing significantly lower viability than controls [19]. To evaluate the cytotoxicity of 3D-printed XL-insert components made from 3D printing materials on SHED cells, we assessed cell viability after exposure times ranging from 0.5 to 72 h.

**Figure 3:**
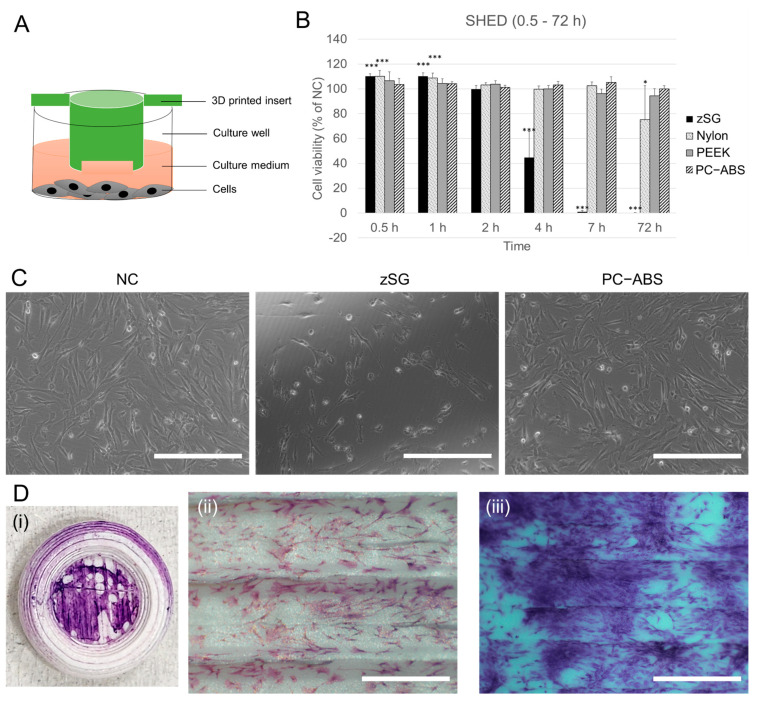
Cytotoxicity to SHED cells of 3D-printed XL-insert components fabricated from different materials. SHED cells (1 × 10^4^ cells/well) were seeded in 24-well plates and cultivated for 24 h. The 3D-printed XL-insert components were then placed in the culture wells above the cells, and cultivation continued for 0.5 to 72 h. At the end of the cultivation period, cell viability was measured using PrestoBlue assays. (**A**) Schematic illustration of the culture model. (**B**) Cell viability of SHED cells. NC, negative control. * *p* < 0.05, *** *p* < 0.001 vs. NC. (**C**) Representative images of SHED cells cultured with 3D-printed XL-insert components fabricated from zSG resin and PC-ABS filament for 4 h. Scale bars = 150 μm. (**D**) SHED cells (0.5 × 10^4^ cells) were seeded in a 3D-printed culture dish (outer diameter = 1 cm) and cultured for 24 to 72 h, followed by Giemsa staining. (**i**) Digital photograph showing SHED cells grown on the 3D-printed PC-ABS culture dish (the STL design file can be found in the Supplemental File S2). (**ii**,**iii**) Microscopic images of SHED cells grown on the surface of the PC-ABS culture dish for 24 h (**ii**) and 72 h (**iii**). Scale bars = 150 μm.

As shown in Figure 3B,C, while zSG resin exhibited strong cytotoxicity after 4, 7, and 72 h of exposure, it did not reduce cell viability within the first 2 h. Interestingly, brief exposure (0.5–1 h) to zSG resin and Nylon 680 significantly stimulated SHED cell growth (*p* < 0.001). However, prolonged exposure (72 h) to Nylon 680 caused a mild reduction in viability (*p* < 0.05). In contrast, PEEK and PC-ABS materials did not adversely affect SHED cell viability at any time point. To further evaluate PC-ABS as a cell culture material, we printed cell culture dishes using PC-ABS filament. Figure 3D demonstrates that SHED cells adhered to the PC-ABS surface and proliferated over time, indicating its suitability for cell culture applications.

### 3.4. PC-ABS Sculptures Promote Osteogenic Differentiation and Gingival Tissue Formation

**Figure 4:**
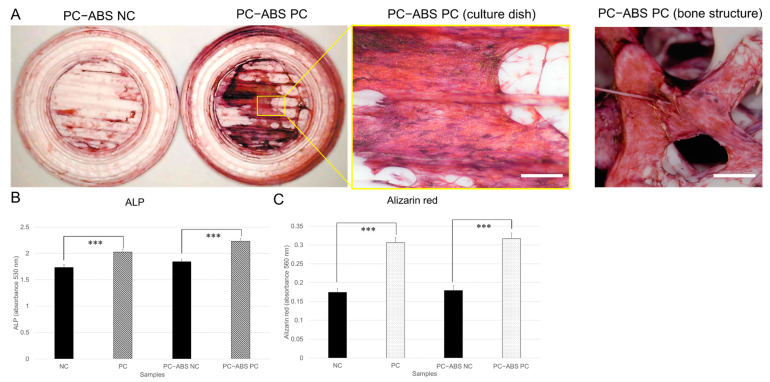
Osteogenic differentiation of SHED cells cultured in 3D-printed PC-ABS culture dishes and micro bone structures. SHED cells (3 × 10^4^ cells/well) were seeded in 3D-printed PC-ABS culture dishes (Supplemental File S2) and micro bone structures (Supplemental File S3) placed in 24-well plates and cultured for 3–4 days. Negative control cells (PC-ABS NC) were maintained in the regular medium, while osteogenic differentiation cells (PC-ABS PC) were cultured in the osteogenic medium for an additional 3 weeks. At the end of the culture period, cells were stained using an ALP staining kit and an Alizarin red solution. (**A**) Representative images of SHED cells cultured on the surface of PC-ABS culture dishes and micro bone structures at the end of cultivation. Red, cells with high ALP activity. Blue, nuclei. Scale bars = 100 µm. (**B**) Quantitative analysis of ALP staining. (**C**) Quantitative analysis of Alizarin red staining. *** *p* < 0.001. Experiments were repeated three times independently.

We further evaluated the effects of PC-ABS sculptures on osteogenic differentiation of SHED cells. As shown in Figure 4A, after 3 weeks of cultivation in the osteogenic medium, SHED cells on PC-ABS culture dishes and micro bone structures (PC-ABS PC) exhibited strong ALP activity compared to the negative control (PC-ABS NC). Quantitative analysis of ALP and Alizarin red staining revealed that SHED cells on PC-ABS sculptures demonstrated significantly higher ALP activity and mineralization capacity, comparable to cells cultured on commercially available 24-well plates (Figure 4B,C).

**Figure 5:**
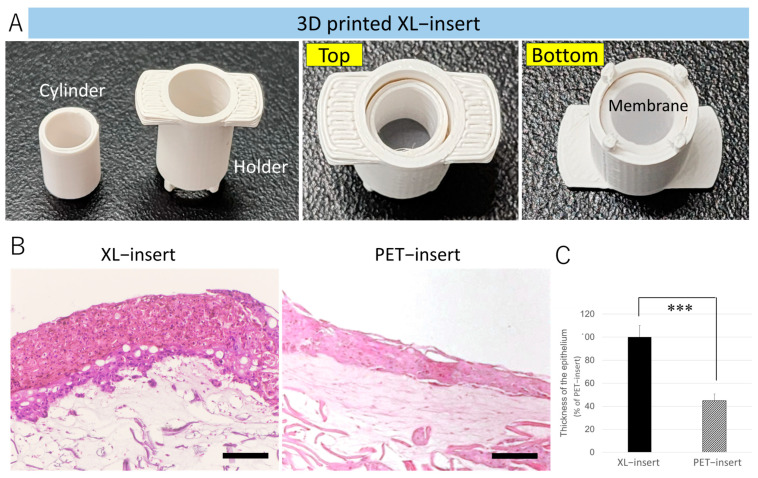
Human gingival tissue formation in 3D-printed PC-ABS XL-inserts. Human gingival tissue equivalents were reconstructed using hGFs and HaCaT cells in a custom XL-insert fabricated with 3D-printed PC-ABS filament. (**A**) Photographs of the 3D-printed components of the XL-insert. The corresponding STL design file is available in Supplemental Files S1 and S4. (**B**) H&E staining of reconstructed human gingival tissue equivalents cultured in a PC-ABS XL-insert or PET-insert. Scale bars = 200 µm. (**C**) Quantification of epithelial thickness using ImageJ software (latest v. 1.54). The histogram illustrates the measured epithelial thickness. *** *p* < 0.001. Experiments were repeated three times independently.

Next, we assessed the suitability of PC-ABS XL-inserts for gingival tissue formation. Using Blender 3.6 software, we designed the XL-insert components, including a cylinder and holder to secure the membrane in place (Figure 5A; Supplemental Files S1 and S4). As shown in Figure 5B, gingival tissue equivalents constructed in the PC-ABS XL-insert displayed a thicker epithelium compared to those in the PET-insert. Quantitative analysis indicated that the epithelial thickness in the PC-ABS XL-insert was 2.2 times greater than in the PET-insert (*p* < 0.001).

## 4. Discussion

This study highlights the significant potential of PC-ABS as a 3D-printable material for dental tissue engineering, addressing critical challenges associated with biocompatibility, cost-effectiveness, and material functionality.

The cytotoxicity of resin-based materials poses a significant challenge for their use in biomedical applications. Consistent with previous studies highlighting the toxic effects of resin monomers like Bis-GMA and TEGDMA [20,21,22], our findings revealed strong cytotoxicity of resin-based 3D-printed components across multiple cell types, including SHED cells, HaCaT keratinocytes, and hGFs. These effects are likely due to the release of reactive oxygen species and unreacted monomers, which induce oxidative stress and apoptosis [23,24]. While the FDA-approved zSG resin demonstrated minimal cytotoxicity within 2 h of exposure, its limited applicability for prolonged use reduces its utility for long-term biomedical applications.

In contrast, thermoplastic filaments, such as PEEK and PLA, are widely explored alternatives for dental applications due to their biocompatibility. However, their mechanical and thermal properties present limitations. PEEK offers excellent mechanical strength and heat resistance but is prohibitively expensive, which hinders its widespread application. PLA, while cost-effective and easy to print, is characterized by weak mechanical properties and low heat resistance, making it unsuitable for demanding dental applications. In contrast, PC-ABS emerges as a balanced alternative, combining the properties of polycarbonate (PC) and acrylonitrile butadiene styrene (ABS) to address these limitations. As shown in Table 1, PC-ABS offers higher mechanical strength and superior heat resistance compared to PLA while being more affordable than PEEK. Our results demonstrated that PC-ABS exhibited no cytotoxic effects even after 72 h of exposure, supporting SHED cell adhesion and proliferation. Additionally, its use in 3D-printed culture dishes and micro bone structures promoted robust osteogenic differentiation, as evidenced by significant ALP activity and mineralization, aligning with prior findings on PEEK and PLA [25,26,27].

PC-ABS offers distinct advantages over both PEEK and PLA. Unlike PEEK, which requires high head temperatures for printing, PC-ABS can be processed at lower temperatures, making it more accessible and compatible with standard 3D printers. Furthermore, PC-ABS is cost-effective and produces sculptures with finer surface finishes, essential for high-precision applications. Compared to PLA, PC-ABS demonstrates superior heat resistance and stronger mechanical properties, making it suitable for applications requiring durability and stability under stress. Its surface properties and mechanical stability likely promote osteoblast-like behavior in SHED cells by mimicking bone-like microenvironments, further enhancing its utility in bone regeneration.

Additionally, our study demonstrated the feasibility of PC-ABS for gingival tissue engineering. Custom-designed PC-ABS XL-inserts supported the formation of gingival tissue equivalents with significantly enhanced epithelial thickness compared to commercially available PET-inserts. This improvement is likely attributed to PC-ABS’s mechanical stability and biocompatible surface properties, which create a conducive microenvironment for epithelial cell proliferation and differentiation. These findings align with previous studies emphasizing the role of material properties in epithelial tissue formation [28,29,30].

Although PC-ABS demonstrated excellent biocompatibility, underscoring its suitability for tissue engineering applications, the PC-ABS sculptures fabricated using standard FDM printing exhibited greater surface roughness compared to resin sculptures. However, this limitation could be addressed by utilizing smaller nozzle diameters or high-precision printers.

## 5. Conclusions

To the best of our knowledge, this study is the first to establish PC-ABS as a versatile and cost-effective material for dental tissue engineering applications. PC-ABS demonstrates excellent biocompatibility and functional performance in promoting cell proliferation, osteogenic differentiation and gingival tissue reconstruction, establishing it as a promising alternative to conventional materials like resins, PLA, and PEEK. By offering an optimal balance of affordability, ease of use, and enhanced material properties, PC-ABS holds significant promise for broader adoption in regenerative dentistry and other biomedical fields.

## Figures and Tables

**Table 1:** 3D printing materials.

Types of Material	Supplier	Heat Resistance of Sculptures (°C)	Tensile Strength (MPa)	Flexural Modulus (MPa)	Young’s Modulus (MPa)	Flexural Strength (MPa)
ABS (acrylonitrile butadiene styrene)-like/Fast resin (ABS resin)	Siraya Tech	135	31.0	N/A	1100	N/A
HARZLabs dental yellow clear resin (SG resin)	HARZ Labs	134	80.0	N/A	N/A	N/A
zSG surgical guide resin (FDA approved) (zSG resin)	Uniz Technology	134	24.8	1242	N/A	48
Nylon680 (FDA approved)	Taulman3D	180	66.0	1670	2220	97
PLA	polymaker	60	28.1	2695	1882	40
PC (polycarbonate)	polymaker	110	59.7	2044	2048	94
PETG	eSUN	80	52.2	1073	N/A	58
PEEK	INTAMSYS	260	99.9	3612	3738	147
PC-ABS	polymaker	135	39.9	2081	1832	66

N/A: not available.

## Data Availability

The data that support the findings of this study are available within the article. Other data related to this study are available on request from the corresponding author.

## Supplementary Materials

The following supporting information can be downloaded at: https://www.mdpi.com/article/10.3390/cells14030167/s1. Supplemental File S1: XL-insert-v15, Supplemental File S2: culture dish 1 cm, Supplemental File S3: Cell culture Bonstructure-3, Supplemental File S4: XL-insert cylinder.
